# Editorial: Genetic and molecular diversity in Parkinson's disease

**DOI:** 10.3389/fnagi.2022.1094914

**Published:** 2022-12-16

**Authors:** Nor Azian Abdul Murad, Siti Aishah Sulaiman, Azlina Ahmad-Annuar, Norlinah Mohamed Ibrahim, Wael Mohamed, Shahrul Azmin Md Rani, Kin Ying Mok

**Affiliations:** ^1^UKM Medical Molecular Biology Institute (UMBI), Universiti Kebangsaan Malaysia (UKM), Kuala Lumpur, Malaysia; ^2^Department of Biomedical Science, Faculty of Medicine, University of Malaya (UM), Kuala Lumpur, Malaysia; ^3^Neurology Unit, Department of Medicine, Faculty of Medicine, Hospital Canselor Tuanku Muhriz, Universiti Kebangsaan Malaysia (UKM), Kuala Lumpur, Malaysia; ^4^Kulliyah of Medicine, International Islamic University Malaysia, Kuantan, Pahang, Malaysia; ^5^Department of Neurodegenerative Disease, University College London (UCL) Institute of Neurology, University College London, London, United Kingdom; ^6^State Key Laboratory of Molecular Neuroscience, Division of Life Science, Molecular Neuroscience Center, The Hong Kong University of Science and Technology, Hong Kong, Hong Kong SAR, China; ^7^Hong Kong Center for Neurodegenerative Diseases, Hong Kong Science Park, Hong Kong, Hong Kong SAR, China

**Keywords:** Parkinson's disease, genetics, diversity, genome-wide association study, Asians

For various reasons, most genetic studies including those related to Parkinson's disease (PD), are primarily performed with populations of European ancestry (Schumacher-Schuh et al., 2022). The insufficient representation of Asian populations in these studies could lead to missed opportunities in novel gene discoveries, drug development, and the advancement of care in multi-ethnic Asian populations. Population-specific differences in PD have been observed in various aspects. For example, epidemiological findings from most studies involving subjects of European ancestry consistently reported a male predominance in PD (Haaxma et al., 2007; GBD 2016 Parkinson's Disease Collaborators, 2018). However, this gender difference is either not apparent or reversed in East Asian studies (Ma et al., 2014; Abbas et al., 2018; Song et al., 2022; Yoon et al., 2022; Zhou et al., 2022).

Similarly, genetic studies have highlighted differences in both monogenic and complex PD between populations. The LRRK2 G2019S variant is more common in Ashkenazi Jewish, North African, and European populations, but extremely rare in East Asian populations, whereas the G2385R variant is only found in East Asian populations (Shu et al., 2019; Simpson et al., 2022). These two variants belong to different functional domains of the LRRK2 protein (Rudenko et al., 2012). While PD pathogenicity may not be related to kinase activity, PD treatment that targets LRRK2 will have to be different in East Asian populations (with a carrier rate of 6–11%) compared to other populations (Simpson et al., 2022). Because of the discrepancy in prevalence across populations, an understanding of variant effects has to be drawn from local populations (Liang et al., 2018; Wang et al., 2022a,b). The GBA gene also poses similar issues. Where there are variants that are only found in one ethnic group but not others (Zhang et al., 2018), there is significant variation (3-31%) in the carrier frequency of GBA gene mutation across populations (Menozzi and Schapira, 2021). The R163Q variant, for example, is regarded as “benign” but mainly exists in East Asian populations. Therefore, in this case, studies confirming the role of pathogenicity should only be conducted in East Asia.

Population variations also play a role in sporadic PD. In one of the largest East Asian genome-wide association studies (GWAS) on PD, Foo et al. replicated some of the same results as studies on European ancestry populations. More importantly, the authors found two novel loci which had not been reported in the European-ancestry GWAS (Foo et al., 2020). The functional significance is yet to be determined, but the findings again point to the need to have a diversity of studies to complete the jigsaw of PD pathogenicity. The same concern applies to atypical Parkinson's disease and other neurological diseases. Taken together with local data, the polygenic risk score may be one such way forward (Sia et al., 2021).

Large GWAS in other Asian populations, particularly people from South East Asia (SEA), is still lacking. A previous meta–genome-wide association study in Asian populations (including SEA Singaporeans and Malaysians), reports similarities and differences in genetic risk factors between Asian and European individuals in the risk for PD, though the study was focused on Han Chinese and South Korean populations (Foo et al., 2020). In fact, a systematic review of all PD publications showed that SEA populations only account for 3% of the total investigated (Schumacher-Schuh et al., 2022). The lack of such underrepresented populations may result in missed opportunities, including the discovery of novel genetic associations for complex traits.

Similarly to East Asian populations, discrepancies in genetic variants associated with PD are observed in SEA populations when compared to people of European and Ashkenazi Jewish ancestry. The LRRK2 G2385R and R1628P are common variants found in East Asia, particularly among the Han Chinese. The G2385R, but not the R1628P variant, is common among Japanese and Korean populations, though the reverse is true for those of Thai ethnicity (Zhang et al., 2017). In Malaysia, both the G2385R and R1628P variants were associated with an increased risk of PD in the Malay and Chinese ethnic groups (Gopalai et al., 2014). More importantly, the N551K variant was protective against PD in Malay individuals (Gopalai et al., 2019), although this finding needs to be replicated in Malay populations from neighboring SEA countries such as Indonesia and the Philippines. Several GBA variants were investigated in multi-ethnic Malaysian populations consisting of Malay, Chinese, and Indian people. The most common variant was L483P, though three novel variants were also identified: P71L, L411P, and L15S. The common European risk variants, E365K, T408M, and N409S, were not detected (Lim et al., 2022). Another study involving only the Malay ethnic group reported that GBA variants may be associated with PD and may modify age of onset amongst the population (Mohamad Pakarulrazy et al., 2020), and this observation was also observed in Thai populations (Pulkes et al., 2014).

Ethnic differences may also play a role in recessive forms of genetic PD. The PINK1 L347P variant, which has not been reported in other populations, had a higher carrier frequency in Filipino people (Rogaeva et al., 2004). A similar observation was also reported recently in Malay ethnic groups (Tan et al., 2020), and two patients of Indian descent in Malaysia (Lim et al., 2021), supporting the pathogenicity of the L347P variant. Another example of population differences is the PINK1 variant identified in Vietnamese individuals with PD, in which the A340T variant was higher in early-onset PD (EOPD, OR = 5.704) (Ton et al., 2020); an opposite association was observed in Han Chinese populations (Wang et al., 2006). Although these findings highlight genetic diversity, most of the studies utilized a small sample size. Thus, larger studies are needed to elucidate the genetic architecture of PD in SEA populations.

In this Research Topic, the need for diversity in PD studies is well-demonstrated. Two EOPD studies in eastern China provide additional information on EOPD in the region. Müller-Nedebock et al. demonstrate an important and interesting point, that even the same mitochondrial DNA variations may have different effects if they are “out of place” of their usual haplotypes. This adds a further dimension of potential variation that scientists and clinicians should study. Besides diversifying genetic studies on PD to complete the jigsaw of PD as far as possible, Akbar et al. also rightly point out that management is also affected by genetics, and discussions and a roadmap on how to address this are needed.

To tackle this missing diversity, a few consortiums were formed in different parts of the world including Africa (Rizig et al., 2021), Central Asia, East Asia (Mok, 2021), Latin America (Zabetian and Mata, 2017), and South Asia (Rajan et al., 2020). Their main aim is to investigate the genetic cause of PD in different populations (Global Parkinson's Genetics Program, 2021). More importantly, these consortiums have started growing from a purely genetic collaboration into a platform for researchers to form new collaborations and address important clinical questions. Ultimately, the better we understand PD, the better our patients will be managed. We should welcome more diversity in the research of PD, and we should actively promote this in the wider community to gain better support.

## Author contributions

NA and SS wrote the manuscript draft. AA-A, NM, WM, and SM reviewed the final manuscript draft. KM wrote and reviewed the manuscript draft. All authors contributed to the article and approved the submitted version.
